# Outbreak Investigations around the World: Case Studies in Infectious Disease Field Epidemiology

**DOI:** 10.3201/eid1511.090931

**Published:** 2009-11

**Authors:** Harry W. Haverkos

**Affiliations:** Author affiliation: Uniformed Services University of the Health Sciences, Bethesda, Maryland, USA

**Keywords:** Epidemiology, case studies, history, infectious disease, bacteria, viruses, parasites, book review

Outbreak investigations are fascinating stories. Mark S. Dworkin, Epidemic Intelligence Service (EIS) Class of 1994, has compiled 19 first-hand accounts of case studies in infectious disease epidemiology and presents them in chronological order. The first is Kenrad Nelson’s 1964 investigation of leptospirosis associated with swimming in an irrigation ditch in rural Washington; the last Patricia Quinlisk’s evaluation of a 2006 mumps epidemic in Iowa. In between are investigations involving 8 bacterial infections, 6 viruses, 1 helminth (*Taenia* *solium*), 1 protozoan (*Cryptosporidium* sp.), and a misdiagnosis of *Entamoeba* *histolytica*. Fourteen of the outbreaks occurred in the United States; of the remaining 5, one each occurred in Portugal, Israel, Egypt, Gabon, and Liberia.

In general, the stories are told as first-person accounts, use an informal style, and include personal reflections. Many chapters, but not all, include epidemic curves, maps, tables, exhibits, and lessons learned. I especially enjoyed reading about Paul Blake’s experience with a cholera outbreak in Portugal, Charles Jennings and measles in Illinois, Daniel Bausch and Ebola in Gabon, and reading both chapters by Jeffrey Davis—toxic shock syndrome and cryptosporidiosis.

As an instructor of epidemiology, I read the book seeking a complementary text for students. The informal style does make enjoyable reading but does not translate into an appropriate textbook. Several of the chapters are too long. One chapter is written by multiple authors, told from 4 points of view, and is very difficult to read. The lessons learned are organized chronologically, not by content. Some lessons are redundant; other areas of epidemiology are not adequately explored, e.g., sampling strategies, study design, questionnaire development and data analysis, population screening, and noninfectious diseases. However, I like the concept of first-hand accounts to supplement epidemiology textbooks. Could one format the chapters as unknowns like that of New England Journal of Medicine case studies? Could outbreak investigations be written in the style of Berton Roueché as medical mysteries, but supplemented with epidemic curves, maps, and lessons learned?

I recommend this book to all infectious disease epidemiologists, EIS officers, and infectious diseases clinicians interested in the aura of outbreak investigations. I also encourage the editor to consider a reformatted second edition to enhance the book’s usefulness as a complementary text in epidemiology coursework.

